# Impact of nutritional diet therapy on premenstrual syndrome

**DOI:** 10.3389/fnut.2023.1079417

**Published:** 2023-02-01

**Authors:** Rodica Siminiuc, Dinu Ţurcanu

**Affiliations:** ^1^Department of Food and Nutrition, Faculty of Food Technology, Technical University of Moldova, Chişinău, Moldova; ^2^Doctoral School of the Technical University of Moldova, Department of Food and Nutrition, Faculty of Food Technology, Technical University of Moldova, Chişinău, Moldova

**Keywords:** premenstrual syndrome, food patterns, nutrients, supplements, menstrual cycle, wellbeing

## Abstract

Premenstrual syndrome (PMS) is one of the most common disorders faced by women of reproductive age. More than 200 symptoms of varying severity associated with PMS have been identified. Because of the broad spectrum of action of PMS and its impact on quality of life, symptom relief is the main challenge of treating PMS and premenstrual dysphoric disorder (PMDD). The review aims to analyze and identify the potential impact of dietary and nutritional therapies on PMS and, respectively, for its better management. The study was conducted by accessing Internet databases such as PubMed, ScienceDirect, and Scopus and using relevant keywords such as PMS, symptoms, dietary patterns (DPs), macro and micronutrients, and supplements. The results showed that diet is an essential modulating factor in reducing and managing PMS symptoms. But research on the actual effect of foods and nutrients on PMS is sparse, sporadic, and studied with insufficient scientific rigor. No correlations were identified between the consumption of macronutrients and PMS: protein, fat, carbohydrates, and fiber, but the effectiveness of micronutrients, especially calcium, magnesium, vitamin D, B vitamins, and herbal supplements, was demonstrated. Researchers remain unanimous that the evidence is insufficient and limited to support their use as an effective treatment. Nevertheless, the results could contribute to providing quality information to help women and girls make evidence-based decisions regarding premenstrual health and the adoption of dietary and nutritional therapies.

## 1. Introduction

**FIGURE 1:**
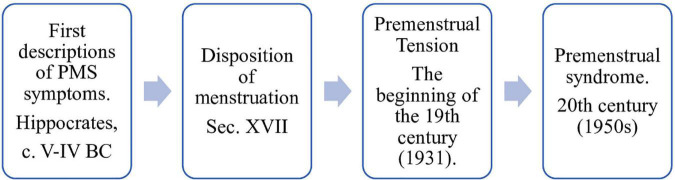
Evolution of the nomenclature for menstrual disorders.

Women’s wellbeing is one of the health’s main goals and is an increasingly good tool for determining the functional impact of some diseases. It also indicates social and economic development and quality of life. The most common problems faced by women, mentioned in the literature, are related to the menstrual cycle (1). Premenstrual syndrome (PMS) is one of the most widespread disorders in reproductive age, negatively impacting women’s emotions, and performance (2). Although the first symptoms, similar to PMS, were described as early as Hippocrates, the diagnostic criteria were specified more recently. Mainly due to the heterogeneity of menstrual symptoms, definitions have varied substantially over the years, evolving from “menstrual moods,” “premenstrual tension,” to “PMS” (3–6). In the late 20s and mid-50s, PMS comes more frequently to the attention of researchers (7) (Figure 1).

PMS is a clinical condition that occurs during the luteal menstrual cycle, that is, during the last 14 days of the menstrual cycle (from ovulation to the onset of menstruation). It is characterized by the cyclic presence of recurrent affective, physical, and behavioral symptoms, which disappear spontaneously within 4 days from the beginning of menstruation and do not recur until at least the day of the cycle (8–11).

Late luteal dysphoric disorder (LLDD), also known as premenstrual dysphoric disorder (PMDD), is the most severe form of PMS. It is considered a medical condition, severely disrupting women’s quality of life, often causing them to seek drug treatment. Currently, PMDD is listed in the Diagnostic and Statistical Manual of Mental Disorders, 5th Edition (DSM-5) as a separate entity under depressive disorders (6, 12).

Due to the poor understanding of the mechanisms underlying PMS, the exact etiology of these premenstrual disorders remains unclear and effective treatments are limited (13). The most well-known hypotheses concerning PMS are associated with hormonal fluctuations, following ovulation, diets with nutritional deficiencies (especially in vitamin B6, magnesium, and calcium), family medical history, which includes depression or anxiety, etc. Symptoms may begin in the early, mid, or late luteal phase and are not associated with defined concentrations of any specific gonadal or non-gonadal hormone (14). Other arguments focus on abnormal serotonergic activity, progesterone, and gamma-aminobutyric acid (GABA) neurotransmitter aberrations (15) and the presumed role of circulating gonadal steroids in the development of symptoms (16, 17). Dibaz and Aksan (16) argue that sex steroids and neurotransmitters play a central role in the etiology of PMS. There is evidence that PMS is twice as pronounced in women with a normal body mass index (BMI) compared to women with a BMI ≥ 25 kg/m^2^ (18).

Women with PMS experience affective or somatic symptoms that cause severe social or occupational dysfunction (14). The range of symptoms associated with PMS is extensive, varying in severity, differentiating from one individual to another, and extending across a range of medical specialties: from gynecological to psychiatric, affecting all aspects of life (11). Over 200 signs and symptoms of PMS have been identified (19). Up to 98% of women report at least one physical and mental symptom before the onset of their menstrual cycle. About 30–40% of women say PMS symptoms that involve drug treatment, and 3–8% of women suffer from PMDD that meets strict DSM-IV criteria (7, 8, 15, 16, 20–24). The most common symptoms are shown in Table 1.

**TABLE 1:** The most common symptoms of premenstrual syndrome (PMS).

Physical symptoms	Psychological and behavioral symptoms
Weight gain	Insomnia/Drowsiness
Edema	Change in appetite: increased appetite
Breast tenderness and swelling	Anxiety and tension
Stomach problems	Decreased libido
Back pain	Depressed mood
Muscle pain	Changes of disposition
Joint pains	State of fatigue
Headaches	Anger
Dizziness	Irritability
Sweating	Weeping
Acne or other skin problems	Restlessness
Constipation or diarrhea	Confusion
Bloating and flatulence	Concentration and memory problems
Cramps	Loss of confidence
Low tolerance to noise and light	Social isolation

The persistence of symptoms tends to fluctuate, with prevalence influenced by cultural and geographic characteristics. For example, France has the lowest PMS rate (12%), and Iran has the highest rate (98%). PMS is not associated with age, educational level, or financial means (8). Depending on the severity of symptoms, PMS can lead to decreased quality of life, reduced occupational productivity, increased dependence on specialized medical care, and interference with interpersonal relationships and daily activities. In addition, PMS may increase the risk of hypertension, negatively impact athletes’ performance and daily activities, and is significantly associated with reduced academic performance (25). The diagnosis of PMS consists of identifying the timing of symptoms about menstruation, the significant change between the severity of post- and premenstrual symptoms, and the significant severity of clinical symptoms (2, 26).

### 1.1. Treatment

Better definitions and research based on strict inclusion-exclusion criteria have allowed the development of effective treatments adapted to the severity of lifestyle disruption and specific individual symptom totals (27). However, due to the broad spectrum of action of PMS and its impact on quality of life, symptom relief is the main challenge of treating PMS and PMDD (28).

This review aims to analyze and identify the potential impact of dietary and nutritional therapies on PMS and, respectively, for its better management.

The study was conducted by accessing Internet databases such as PubMed, ScienceDirect, and Scopus and using relevant keywords such as PMS, symptoms, dietary patterns (DPs), macro and micronutrients, and supplements.

## 2. Impact of dietary patterns and macronutrients on PMS

Although people who experience severe PMS symptoms often require medical intervention, most women repress them without diagnosis or management. To date, no treatment is universally recognized as effective, and many women seeking relief often turn to therapeutic approaches outside of conventional medicine (29, 30).

Diet appears to be an essential modulating factor in reducing and managing some of the symptoms of PMS. But the actual effect that foods and nutrients have on women with menstrual disorders is not studied with enough scientific rigor (2, 31). It is recommended to follow a healthy food model, in which fresh, unprocessed foods predominate and avoid those rich in carbohydrates or refined fats, salt, alcohol, and stimulant drinks.

Following a healthy diet and managing stress are important factors in preventing and managing PMS (32).

In a study that looked at the impact of three DPs: traditional DP, high in eggs, tomato sauce, fruit, and red meat; healthy DP, rich in dried fruits, spices, and nuts and Western DP, characterized by high consumption of fast food, carbonated drinks, and processed meat. Western DPs were positively associated with PMS, while healthy and traditional nutritional habits had an inverse correlation (33). Research has shown that short-term intermittent fasting can lead to more excellent parasympathetic activity and lower luteal cortisol levels in young women. These results indicate the possibility of producing an anti-stress effect in the luteal phase, which would reduce menstrual symptoms (34, 35).

It has been suggested that caloric intake, as well as preferential carbohydrate selection, during the premenstrual period is more significant in women with PMS, who are considered to be more sensitive to cyclical hormonal or neurotransmitter fluctuations (36). The improvement in mood after carbohydrate ingestion is explained by the increase in serotonin associated with tryptophan, ameliorating a potentially functional deficiency of serotonin in the brain and thus serving as self-medication. At the same time, a diet with excess sugars, especially simple fats, fried foods, coffee, and alcohol, correlates positively with the development of PMS. In order to reduce PMS symptoms, the authors recommend a diet rich in vegetables, fruits, and healthy fiber (23, 37–39).

Other research, which studied the impact of macronutrient intake on PMS, reported that no correlation was found between the consumption of protein, fat, carbohydrates, fiber, and PMS. But it is suggested that maltose might be associated with PMS (37, 40), and high intake of stearic acid may be associated with a lower risk of developing PMS. Further prospective research is needed to confirm this finding (23).

Tests were carried out on subjects who followed diets in which 40% of energy came from fat, alternated with periods of the regime with only 20% of energy from fat. The subjects were randomized into two categories: one category that had a ratio of polyunsaturated and saturated fatty acids of 1.0 and another group–with a ratio of 0.3. There were no significant differences in self-reported menstrual symptoms between the two groups (polyunsaturated/saturated), but significant decreases in symptoms associated with water retention were reported (41). Total fat intakes (saturated and monounsaturated) were significantly correlated with pain symptoms (42).

## 3. Impact of micronutrients and herbal supplements

Zinc is known to have multiple beneficial effects, including anti-inflammatory, antioxidant, and antidepressant actions. Overall, zinc supplementation for 12 weeks among women with PMS had a beneficial impact on physical and psychological symptoms, total antioxidant capacity, and brain-derived neurotrophic factor. However, data on the effects of zinc supplementation on biomarkers of inflammation, oxidative stress, and antidepressant impact among young women with PMS are scarce (43).

Several studies have shown that subjects with PMS have lower serum calcium levels, and calcium supplementation could significantly improve the incidence of PMS and its associated symptoms (44–47). However, further clinical studies are needed to establish a firm link between calcium and PMS (48). Other research has justified the approach of a high intake of calcium associated with vitamin D in reducing PMS symptoms, including lowering the risk of osteoporosis and some cancers (49). Calcium and vitamin D supplementation is recommended as an inexpensive, low-risk, acceptable, and accessible approach to eliminate or reduce symptoms (50). Still, it is not known whether these nutrients can prevent the initial development of PMS (49). Various studies show the importance of vitamin D in female reproduction, probably due to its effects on calcium homeostasis, cyclic sex steroid hormone fluctuations, or neurotransmitter function (51–54). It also helps reduce dysmenorrhea, inflammation, and antioxidant markers in women with PMS and vitamin D deficiency (55, 56). In adolescents, vitamin D therapy is associated with improvements in PMS-related quality of life and mood disorders (57). There is also research showing that vitamin D supplementation for 12 weeks had no significant impact on other PMS symptoms (58).

Magnesium supplementation is considered effective in preventing dysmenorrhea, PMS, and menstrual migraine (59). A combination of magnesium with vitamin B6 can effectively reduce premenstrual stress, and vitamin B6 can effectively reduce anxiety in older women (60).

Thiamine (B1), riboflavin (B2), niacin (B3), pyridoxine (B6), folic acid (B9), and cobalamin (B12) are indispensable vitamins in the synthesis of neurotransmitters potentially involved in the pathophysiology of PMS (61). Research on the impact of dietary intake of niacin, pyridoxine, folate, and cobalamin on PMS has not shown significant associations. Intake of B vitamins from supplements was not associated with a lower risk of PMS. But, a significantly lower risk of PMS was observed in women with a high intake of thiamin and riboflavin from food sources (61).

A comparative study, with reference to the effectiveness between vitamin B6 and broad-spectrum micronutrient formulas (which included minerals and vitamins) on PMS showed that both treatments provided similar benefits: the micronutrient formulas had a more significant effect on the quality of life, as well as a potential clinical use for PMDD. However, vitamin B6 therapy appears to be as effective as broad-spectrum formulas (62). In another study, which looked at the impact of 62 herbs, vitamins, and minerals on PMS, only calcium intake was justified in reducing PMS. The authors argue that further research, with sufficient sample sizes and measuring the effect on individual PMS symptom severity, is needed (30) to support the use of calcium, vitamin D, and vitamin B6 supplements, as well as herbal remedies. Evidence supporting cognitive behavioral therapy is also insufficient (28, 63).

Nor is the effectiveness of dietary supplements sufficiently researched. Although some research claims that neither evening primrose oil nor St. John’s wort has any different effect than placebo (29), other research shows the positive impact of evening primrose oil on PMS (32). The potential beneficial effect of curcumin in alleviating the severity of PMS symptoms, possibly mediated by curcumin’s neurotransmitter modulation and anti-inflammatory effects, is also noted (64, 65). Jafari et al. (66) highlights the potential impact of garlic in reducing the severity of PMS and its possible use as an alternative therapy in the prophylaxis and treatment of premenstrual disorders.

Limited evidence supports the promotion of a healthy diet, exercise, and vitamin supplementation in reducing PMS, but their advertising is recommended for their apparent health benefits in general. Lifestyle modification and regular exercise may have a more pronounced positive effect in milder cases of PMS (16). At the same time, it is recommended to minimize the intake of salt, caffeine, and tobacco (15, 67–69). A study of students in the United Arab Emirates reported that fruit consumption was associated with a reduced risk of behavioral symptoms, and smoking and consumption of caloric foods (high in fat, sugar, and salt) were identified as risk factors vital for PMS (25).

## 4. Conclusion

• The range of symptoms associated with PMS is extensive, with varying severity, extending to most aspects of women’s lives, and requiring knowledge, monitoring, and a personalized approach to diagnosing psychological and physical conditions.
• The best-known hypotheses, which explain the causes of PMS, are associated with hormonal fluctuations and nutritional deficiencies, especially in vitamin B6, magnesium, and calcium.
• To date, no treatment is universally recognized as effective, and many women seeking relief often turn to therapeutic approaches outside of conventional medicine.
• Diet is an essential modulating factor in reducing and managing PMS symptoms. But research on the actual effect of foods and nutrients on PMS is sparse, sporadic, and studied with insufficient scientific rigor.
• The study did not identify correlations between the consumption of macronutrients: proteins, fats, carbohydrates, fibers, and PMS. But it is suggested that maltose might be associated with PMS.
• Some studies have shown the effectiveness of micronutrients, especially calcium, magnesium, vitamin D, B vitamins, and herbal supplements, in reducing PMS. But researchers agree that the evidence is insufficient and limited to support their use as an effective treatment.
• Lifestyle, nutrition, and general health considerations appear to be essential strategies in the reduction or management of menstrual symptoms but are recommended to be promoted more for their apparent health benefits than as conclusive evidence for reducing negative experiences of PMS.
• Awareness of health and nutrition professionals to inform the public about the complexity of factors influencing PMS and the need for training/education regarding self-care practices for PMS management is current and necessary. At the same time, nutrition service providers should approach and adapt dietetic-nutritional therapy in a personalized way to reduce PMS. In addition, to have and provide quality information to help women, including young women/adolescents, make evidence-based decisions about premenstrual health and the adoption of diets, nutrients, or supplements.

## Author contributions

Both authors listed have made a substantial, direct, and intellectual contribution to the work, and approved it for publication.
